# Transposon debris in ciliate genomes

**DOI:** 10.1371/journal.pbio.3001354

**Published:** 2021-08-24

**Authors:** Yi Feng, Laura F. Landweber

**Affiliations:** Departments of Biochemistry & Molecular Biophysics and Biological Sciences, Columbia University, New York, New York, United States of America

## Abstract

Ciliate genomes are replete with "junk" DNA insertions that require DNA splicing for their removal. What is the evolutionary source of these insertions? This Primer explores a new study in PLOS Biology that supports a classic model of transposon origins.

## Teaser

The germline genomes of ciliated protists are replete with “junk” DNA insertions that need to be removed for gene expression. Unlike introns, these are spliced as DNA. What is their source, and why are they so abundant? A new study in *PLOS Biology* supports a classic model of transposon origins.

A genome encodes all the primary information that DNA-based organisms need through their life span. Although it has been assumed to be identical in most cells of Metazoa, some animals, such as nematodes, sea lampreys, and songbirds, contain multiple genomes that usually differ between germline and somatic cells [1]. Another example of somatic rearrangement is V(D)J recombination in jawed vertebrates, which permits the production of diverse antibodies and receptors for adaptive immunity [1]. In addition to these examples in Metazoa, ciliates, which are microbial eukaryotes, contain 2 distinct genomes packaged in a somatic macronucleus (MAC) and a germline micronucleus (MIC) within the same cytoplasm. Only the MAC genome is transcriptionally active during asexual (vegetative) growth. During postzygotic development, the MAC genome rearranges from a copy of the MIC genome by deleting internal eliminated sequences (IESs) and other repetitive regions from the DNA, together with rejoining of flanking MAC-destined segments. Although analogous to RNA splicing, these rearrangements occur on DNA. *Paramecium tetraurelia* removes approximately 45,000 IESs during genome differentiation. Most interrupt coding regions [2]. Therefore, precise elimination is necessary for proper gene function. This gives rise to a long-standing question in the field: How did IESs accumulate if their presence is deleterious?

A study published in the current issue of *PLOS Biology* [3] provides evidence to support a model proposed by Klobutcher and Herrick in 1997 that linked the origin of IESs to transposable elements (TEs) in ciliate genomes [4]. TEs play a key role in genome rearrangement in other cells and are occasionally domesticated by the host. A classic example is the origin of RAG1 recombinase by domestication of a *Transib* transposase [2]. In humans, mutations in the PiggyBac TE-derived 5 (PGBD5) gene trigger aberrant genome rearrangement in some childhood cancers, emphasizing the importance of regulating genes derived from transposon domestication [5]. In ciliates, multiple lines of evidence support the role of TE-derived genes in DNA elimination. In *Oxytricha trifallax*, over 1 billion years diverged from *Paramecium*, tens of thousands of MIC-limited TEs from the *Tc1/mariner* family are expressed during development, and silencing of transposase genes via RNA interference (RNAi) stalls DNA elimination [6]. The *Paramecium* and *Tetrahymena* lineages domesticated transposase genes from the piggyBac family, and their silencing also prevents correct DNA elimination [7,8]. Further, an analysis of 20 *Paramecium* IESs suggested that they could be decayed transposons [9]. The IESs share an 8-bp consensus 5′-TAYAGYNR-3′ on the boundary, similar to sequences flanking *Tc1/mariner* transposons [9]. Based on these observations, it is necessary to revisit the Klobutcher and Herrick model.

**Fig 1:**
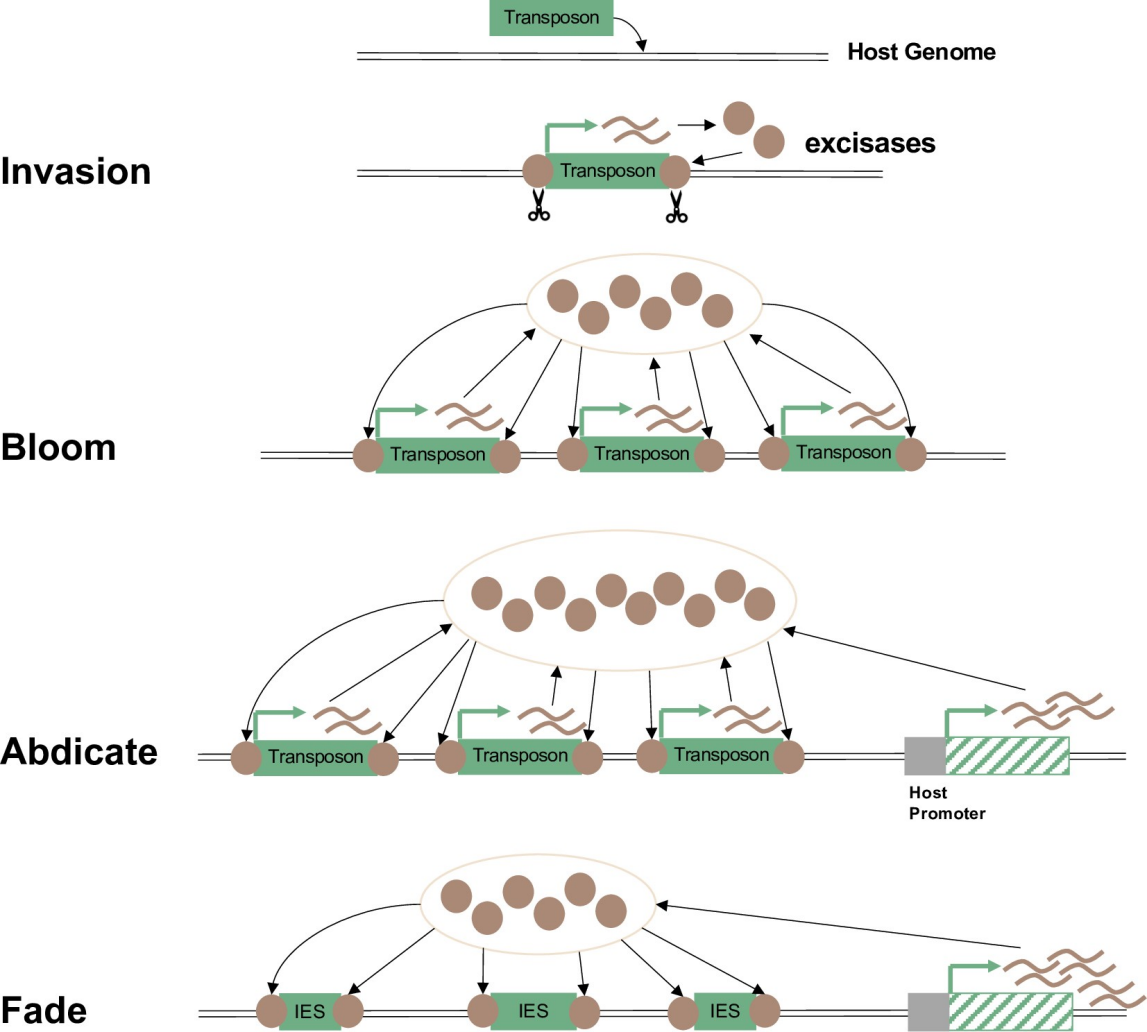
Klobutcher and Herrick’s original proposal [4] that most IESs are the decayed remnants of transposons. This model is abbreviated as “IBAF” to describe the gradual conversion of transposons into IESs in 4 steps: invasion, bloom, abdicate, and fade. Invasion: One or more transposons, perhaps of the *IS630-Tc1-mariner* superfamily (green box), invaded the host ciliate germline genome, and many transposons may still be active to this day. Active transposons encode a transposase (brown circles) that permits their excision from or movement within the genome. Bloom: Transposon expansion via replicative transposition across the genome. Their encoded transposase/excisase proteins, capable of acting on each other, become a common good, contributing to the excision of transposons other than one’s self. Abdicate: A gene (green hashed box) that encodes the excisase becomes placed under a host promoter for stable and regulated expression, but loss of mobility. In some cases, this gene may also be retained in the MAC (e.g., PiggyMac in *Paramecium tetraurelia*). Fade: Mutation accumulation in most transposons gradually converts them into short IESs, nonautonomous elements that retain motifs to permit their recognition and cleavage from the genome by host excisases. IBAF, invasion, bloom, abdicate, and fade; IES, internal eliminated sequence; MAC, macronucleus; MIC, micronucleus.

Like a whimsical M.C. Escher metamorphosis print, the model describes 4 stages to transform transposons into IESs: invasion, bloom, abdicate, and fade or “IBAF” (Fig 1). An autonomous transposon first invades the host genome, bringing with it its encoded transposase to cut and paste itself. Once there are enough transposon insertions in gene regions, it would become beneficial for the host to permit sufficient translation of transposase protein to excise these insertions. The germline MIC provides a safe haven for transposons to flourish (bloom) as long as they are safely eliminated before the formation of the MAC. Indeed, modern ciliate germline genomes may still be prone to transposon invasion, but the trait of ciliate nuclear dimorphism itself may have evolved as an evolutionary strategy to corral them. Some hosts could eventually domesticate or repurpose a transposase gene by placing it under control of their own promoters, which would relax the selection pressure on the transposase ORF encoded in TEs, hence abdicating their role as excisase and allowing more mutations to accumulate across the crippled TE. The decayed TEs may rapidly atrophy to short IESs and lose the protein-coding ability (fade), as long as their boundary sequences can still be recognized and removed efficiently. While this model may explain the origin of IESs, there has been mostly indirect evidence to support it (e.g., the resemblance of sequences flanking IESs to TE target site motifs [10]). Sellis and colleagues provide compelling evidence that thousands of IESs derive from TEs by using comparative genomics within the *Paramecium* genus [3].

In this study, Sellis and colleagues annotated and dated IESs in 9 diverse species of *Paramecium* [3]. They sequenced both genomes in each species and annotated IESs by comparing short MIC reads to the MAC genome assemblies. IESs were classified as old, intermediate, or recent on a phylogeny. To explore whether IESs derived from TEs, they clustered IESs to reveal shared ancestry. While the majority of IESs are unique, 24 families contained at least 10 homologous IESs across the *Paramecium* species. Notably, 4 families are homologous to the *IS630-Tc1-mariner* transposon superfamily. Moreover, 97.5% of these clustered, mobile IESs seem to be recent insertions, much higher than the 9.5% of overall IESs. This is expected, as older IESs accumulate substitutions, obscuring homology. Two species of different subclades, *Paramecium sonneborni* and *Paramecium tredecaurelia*, were found to share several mobile IES families, likely the result of horizontal gene transfer.

Old IESs are less often under epigenetic control than new ones [3]. In *P*. *tetraurelia*, 70% of IESs require histone methylation or small RNAs for removal, while the rest are independent of either pathway [11]. The length distribution also differs between older and newer IESs. Older IESs are much shorter, compatible with the “fade” step of the model. Moreover, some conserved IESs encode MIC-limited genes [3,12] or provide a “switch” to regulate gene expression.

This study demonstrates a workflow for studying IES evolution within a lineage. In the future, it will be illuminating to compare mobile IES families across clades. Unlike the consensus “TA” flanking IESs in *Paramecium*, IESs in some other lineages are flanked by diverse microhomologous sequences, although the shortest repeats still resemble transposon ends or target site duplications [10], harkening back to likely transposon origins. Long-read genome assemblies may provide a more complete portrait of transposon–IES evolution in ciliates.

Sellis and colleagues suggest that IESs dispersed across the genome within a clade may derive from TEs. Identifying the initial invading TE insertions is an open question in the IBAF model. This model is also similar to the one proposed for intron evolution. Recent evidence demonstrated that DNA transposons have generated hundreds to thousands of introns in 2 species separated by 1 billion years [13]. Both programmed DNA deletion and intron splicing offer strategies to remove harmful insertions from functional genes. This opportunity for accurate deletion assuages their deleteriousness, permitting a stealth bloom of TE insertions, which may also beget innovation, permitting the evolution of domestication [7,8] or mutualism [2,6] between host and genomic parasite.

## Abbreviations

MAC: macronucleus
MIC: micronucleus
IES: internal eliminated sequence
TE: transposable element
PGBD5: PiggyBac TE-derived 5
RNAi: RNA interference
IBAF: model, invasion, bloom, abdicate, fade
